# ﻿The genus *Neurigona* Rondani, 1856 (Diptera, Dolichopodidae) from Yunnan, China, with descriptions of seven new species and a key to the species of China

**DOI:** 10.3897/zookeys.1190.109222

**Published:** 2024-01-31

**Authors:** Chen Lin, Mengqing Wang, Ding Yang

**Affiliations:** 1 Institute of Life Science and Technology, Inner Mongolia Normal University, Huhhot, 010022, China China Agricultural University Beijing China; 2 Department of Entomology, College of Plant Protection, China Agricultural University, Beijing, 100193, China Inner Mongolia Normal University Huhhot China; 3 Institute of Plant Protection, Chinese Academy of Agricultural Sciences, Beijing, 100193, China Institute of Plant Protection, Chinese Academy of Agricultural Sciences Beijing China

**Keywords:** Identification key, long-legged fly, morphology, Neurigoninae, new taxon, Oriental Realm, sympatric species richness

## Abstract

Previously, only three species of the genus *Neurigona* Rondani of the subfamily Neurigoninae were known from Yunnan Province. Here, we reviewed the species of *Neurigona* from Yunnan and added the following seven new species: *N.apicilata***sp. nov.**, *N.basicurva***sp. nov.**, *N.brevidigitata***sp. nov.**, *N.convexa***sp. nov.**, *N.huanglianshana***sp. nov.**, *N.quadrimaculata***sp. nov.**, and *N.ventriprocessa***sp. nov.** All seven new species are sympatric and were collected from below a reservoir in the Huanglianshan Nature Reserve in Yunnan using three Malaise traps in 2019. This suggests a very high species richness in the Yunnan fauna. A key to the species of *Neurigona* from Chinese mainland is provided.

## ﻿Introduction

The genus *Neurigona* Rondani, 1856 belongs to the subfamily Neurigoninae within Dolichopodidae. They usually have non-metallic yellow coloration on much of the thorax, legs, and abdomen, and can be separated from other Dolichopodidae genera by the following features: proboscis with a pair of long and pale ventral hairs; posterior mesonotum distinctly flattened; legs slender and long, and anterior preapical setae on mid and hind femora absent; sternite 5 sometimes with a ventral projection in males; fore tarsomere 1 usually elongated; vein M gently or strongly bent apically and convergent with R_4+5_; surstylus divided into dorsal and ventral lobes; cercus with digitiform internal median projection (Naglis 2003; Kazerani et al. 2022). Adults of most species are regularly observed on tree trunks where the males also exhibit their courtship behavior, sometimes in large numbers (Parent 1938).

*Neurigona* is the most speciose genus of the Neurigoninae with 166 known species from the world, of which 44 species are from the Palaearctic, 44 from the Neotropical, 39 from the Nearctic, 32 from the Oriental, four from the Australian, and three from the Afrotropical realms (De Meijere 1916; Becker 1922; Parent 1935, 1944; Dyte 1975; Negrobov 1987, 1991; Negrobov and Fursov 1988; Pârvu 1996; Yang 1999; Yang and Saigusa 2001a, b, 2005; Naglis 2003; Zhang et al. 2003; Wang et al. 2006, 2007, 2010, 2020; Yang et al. 2006, 2011; Grichanov 2010; Capellari 2013; Grootaert and Foo 2019; Da Silva et al. 2022; Kazerani et al. 2022). Thirty-one species were known to occur in China (Wang et al. 2010; Yang et al. 2006, 2011, 2018).

Yunnan Province, located in southwestern China, boasts a rich and diverse ecological environment, such as tropical rainforests, subtropical forests, alpine meadows and wetlands. Its eastern part is the Yunnan-Guizhou Plateau, which is one of the four major plateaus in China, and its northwest part is the Hengduan Mountains, which is the longest and widest north-south mountain range in China (Feng et al. 2023). The presence of multiple habitat types within a relatively small geographical area enhances species diversity. With its breathtaking landscapes and abundant biodiversity, it is considered one of the most ecologically significant regions in China. Thus far, only three species, *N.centralis* Yang & Saigusa, *N.ventralis* Yang & Saigusa, and *N.yunnana* Wang, Yang & Grootaert, were recorded from Yunnan (Yang et al. 2006, 2018). In this paper, seven new species of *Neurigona* are added to the fauna of Yunnan, and a key to the males of species of *Neurigona* in Chinese mainland is provided.

## ﻿Material and methods

The specimens on which this study is based were collected from below the reservoir in the Huanglianshan National Nature Reserve in Yunnan using three Malaise traps in 2019. All specimens are deposited in the Entomological Museum of China Agricultural University (**CAU**), Beijing. The morphological terminology for adult structures, including the structures of the male genitalia, follow Cumming and Wood (2017). All the figures were taken and drawn by Chen Lin. The following abbreviations are used:

acr acrostichal bristle (s);

**ad** anterodorsal bristle (s);

**av** anteroventral bristle (s);

**dc** dorsocentral bristle (s);

**pd** posterodorsal bristle (s);

**pv** posteroventral bristle (s);

**v** ventral bristle(s);

**CuAx ratio** length of dm-cu / length of distal portion of CuA;

LI fore leg;

LII mid leg;

**LIII** hind leg.

## ﻿Taxonomy

### ﻿Key to species (males) of *Neurigona* from Chinese mainland (modified from Yang et al. 2011 and Wang et al. 2010)

**Table:** 

1	Abdominal sternite 5 not projected	**2**
–	Abdominal sternite 5 projected, subtriangular or subquadrate	**17**
2	Fore coxa with wholly black bristles on antero-apical portion	**3**
–	Fore coxa partly or entirely with yellow apical bristles on antero-apical portion	**8**
3	Fore tarsomere 1 shorter than fore tibia	**4**
–	Fore tarsomere 1 longer than fore tibia	**7**
4	More than 10 paired acr bristles	**5**
–	Four irregularly paired acr bristles	***N.yunnana* Wang, Yang & Grootaert, 2007**
5	17–18 paired acr bristles; fore tibia with 1 ad bristle	***N.concaviuscula* Yang, 1999**
–	11–12 paired acr bristles; fore tibia without ad bristles	**6**
6	Fore tarsomere 4 with basal 1/2 swollen and apical 1/2 concave ventrally, fore tarsomere 5 with basal 1/4 swollen and middle portion concave ventrally (Wang et al. 2010: fig. 6)	***N.sichuana* Wang, Chen & Yang, 2010**
–	Fore tarsomeres 4 and 5 normal, without swollen or concave portion	***N.hainana* Wang, Chen & Yang, 2010**
7	Fore femur without row of av bristles; mid and hind tibiae wholly yellow; epandrium with two long and wide lateral processes; ventral lobe of surstylus straight and broad, apically with 4 strong hairs (Yang et al. 2011: fig. 999c)	***N.guangdongensis* Wang, Yang & Grootaert, 2007**
–	Fore femur with row of short av bristles; mid and hind tibiae each with black apex; epandrium with two slender lateral processes; ventral lobe of surstylus curved and slender, apically with 1 thick hair (Yang et al. 2011: fig. 991b)	***N.bimaculata* Yang & Saigusa, 2005**
8	Fore femur with row of yellow av bristles	***N.shennongjiana* Yang, 1999**
–	Fore femur without row of yellow av bristles	**9**
9	Mesonotum with black mid-posterior or lateral spot(s)	**10**
–	Mesonotum entirely yellow	**13**
10	Fore tarsomere 5 slightly thickened with long and strong bristles	**11**
–	Fore tarsomere 5 not thickened, without long and strong bristles	**12**
11	Pleuron chiefly yellow; fore claw distinctly elongated, spine-like	***N.micropyga* Negrobov, 1987**
–	Pleuron chiefly black; fore claw normal	***N.yaoi* Wang, Chen & Yang, 2010**
12	Postnotum wholly blackish; mid tibia and tarsus without row of short dense crochet-like av hairs, with only mid tarsomere 1 with row of erect av hairs	***N.centralis* Yang & Saigusa, 2001**
–	Postnotum yellow with a nearly W-shaped dark brown spot; mid tibia and tarsus with row of short dense crochet-like av hairs	***N.brevidigitata* sp. nov.**
13	Fore tibia without ad or pd bristle; hind tarsomere 1 without ventral bristle at base; wing wholly hyaline	**14**
–	Fore tibia with one ad bristle and one pd bristle; hind tarsomere 1 with ~ 15 short erect spine-like black ventral bristles at base; wing with anterior apical corner brownish	**15**
14	First flagellomere brown (Fig. 8A); fore tarsus not distinctly shortened	***N.apicilata* sp. nov.**
–	First flagellomere brownish with yellow base; fore tarsomeres 3–4 shortened, each 0.4× and 0.2× as long as tarsomere 2 respectively	***N.ventralis* Yang & Saigusa, 2005**
15	Fore tarsomere 1 not shorter fore tibia; mid tarsomere 1 not shorter than mid tibia	**16**
–	Fore tarsomere 1 shorter than fore tibia, 0.8× as long as fore tibia; mid tarsomere 1 shorter than mid tibia, 0.9× as long as mid tibia	***N.xiaolongmensis* Wang, Yang & Grootaert, 2007**
16	Fore tarsomere 1 as long as fore tibia; mid tarsomere 1 longer than mid tibia, 1.4× as long as mid tibia; ventral surstylus rather wide (Yang et al. 2011: fig. 1005c)	***N.qingchengshana* Yang & Saigusa, 2001**
–	Fore tarsomere 1 longer than fore tibia, 1.1× as long as fore tibia; mid tarsomere 1 as long as mid tibia; ventral surstylus relatively narrow (Fig. 6B)	***N.quadrimaculata* sp. nov.**
17	Abdomen metallic green with basal part yellow	**18**
–	Abdomen mainly yellow with black spot(s)	**19**
18	Abdomen metallic green, tergum 1 with yellow basal margin; all legs yellow	***N.grisea* Parent, 1944**
–	Abdominal segments 1–3 yellow; legs chiefly yellow except brownish mid coxa and brown to black from hind femur to hind tarsus	***N.xui* Zhang, Yang & Grootaert, 2003**
19	Mesonotum entirely yellow	**20**
–	Mesonotum with small or large mid-posterior area brown or black	**27**
20	Fore tarsomere 4 with basal half swollen	**21**
–	Fore tarsomere 4 with no swelling	**22**
21	Arista dorsal (Yang et al. 2011: fig. 1010a); five dc bristles; 11–12 paired acr bristles; dorsal lobe of surstylus with apical incision (Yang et al. 2011: fig. 1010c)	***N.xiangshana* Yang, 1999**
–	Arista apical (Yang et al. 2011: fig. 1001b); eight dc bristles; 17–18 paired acr bristles; dorsal lobe of surstylus without apical incision (Yang et al. 2011: fig. 1010c)	***N.guizhouensis* Wang, Yang & Grootaert, 2007**
22	Fore tarsomere 1 shorter than or as long as fore tibia	**23**
–	Fore tarsomere 1 distinctly longer than fore tibia, 1.1× as long as fore tibia	**26**
23	Scutellum with small brownish basal spot; fore tarsomere 1 as long as fore tibia	***N.basalis* Yang & Saigusa, 2005**
–	Scutellum without basal spot; fore tarsomere 1 shorter than fore tibia, between 0.7 to 0.9× respectively	**24**
24	Fore coxa with six to seven black anterior apical bristles; dorsal lobe of surstylus with short and obtuse process at dorsal corner (Yang et al. 2011: fig. 1006b)	***N.shaanxiensis* Yang & Saigusa, 2005**
–	Fore coxa with four to six yellow apical bristles; surstylus not as above	**25**
25	Mid coxa with black hairs and bristles; dorsal lobe of surstylus with long and thin process at dorsal corner (Yang et al. 2011: fig. 1002c)	***N.henana* Wang, Yang & Grootaert, 2007**
–	Mid coxa with yellow hairs and bristles; dorsal lobe of surstylus without process at dorsal corner (Yang et al. 2011: fig. 1009c)	***N.wui* Wang, Yang & Grootaert, 2007**
26	Six dc bristles; 18–19 paired acr bristles; dorsal lobe of surstylus short and wide (Yang et al. 2011: fig. 1011b)	***N.xizangensis* Yang, 1999**
–	Three dc bristles; acr bristles absent; dorsal lobe of surstylus with wide base and long obtuse apex (Yang et al. 2011: fig. 1003c)	***N.jiangsuensis* Wang, Yang & Grootaert, 2007**
27	Abdominal sternite 5 subtriangular; fore tibia not thickened and bent at base	**28**
–	Abdominal sternite 5 quadrate (Fig. 9A); fore tibia weakly thickened and bent at base	***N.basicurva* sp. nov.**
28	Wing normal at anterior margin; first flagellomere not triangular	**29**
–	Wing subapically convex at anterior margin (Fig. 4B); first flagellomere somewhat triangular (Fig. 8D)	***N.convexa* sp. nov.**
29	10–15 paired acr bristles	**30**
–	18–19 paired acr bristles	**31**
30	Frons and face yellow; first flagellomere quadrate (Fig. 8G); mesonotum brownish to brown, but notopleuron somewhat pale; fore tarsomere 3 shortened and tarsomere 4 ventrally weakly concave with mammillary ventral process at extreme base; M strongly bent, strongly geniculate, nearly in a right angle; abdominal sternite 5 weakly projected, short subtriangular (Fig. 9D)	***N.ventriprocessa* sp. nov.**
–	Frons and face wholly metallic green; first flagellomere somewhat oval (Fig. 8E); mesonotum with brown subtriangular spot at middle posterior region; fore tarsus not modified; M distinctly bent, somewhat geniculate, in an obtuse angle; abdominal sternite 5 distinctly projected, long subtriangular (Fig. 9C)	***N.huanglianshana* sp. nov.**
31	Mesonotum with narrow brown mid-posterior spot; mid tibia and tarsus with row of short erect pale av bristles; cercus with long apical process (Yang et al. 2011: fig. 1016b)	***N.zhejiangensis* Yang, 1999**
–	Mesonotum with wide black mid-posterior area; mid tibia and tarsus without row of av bristle; cercus without apical process (Yang et al. 2011: fig. 1000b)	***N.guangxiensis* Yang, 1999**

#### ﻿ Neurigona apicilata sp. nov.

Taxon classificationAnimaliaDipteraDolichopodidae

AD6023DA-FC70-5880-8504-5CEAFFC2D2F1

https://zoobank.org/DDF79F46-4E9A-48E7-B6C7-1EF3B7893122

Figs 1 , 8

##### Type material.

***Holotype***: ♂, **China**: Yunnan, Lvchun, Yakou, Shuikuxiafang, (23°3'25.2"N, 102°46'37.71"E), 1780 m, collected by Malaise trap, 2019.IV.19-V.19, Liang Wang and Xin Li (CAU). ***Paratypes***: 19 ♂♂, the same data as holotype (CAU).

##### Diagnosis.

Male eyes contiguous on face. Mesonotum wholly dark yellow; scutellum dark yellow; postnotum dark brown posteriorly. Laterotergite with two minute dark brown or black spots at anterior margin and one large dark brown spot posteriorly. Hind tarsomere 1 with cluster of short thick posterior bristles at extreme base, several short ventral bristles and four short apical bristles (MSSC). Ventral surstylus rather wide, widened apically.

##### Description.

**Figure 1.**
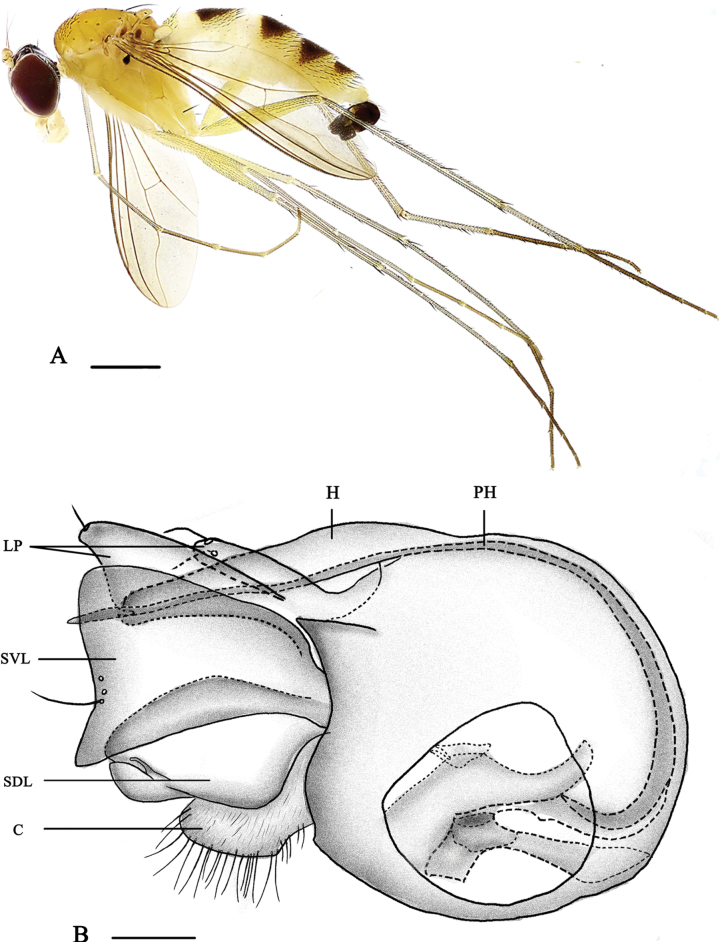
*Neurigonaapicilata* sp. nov. **A** male habitus, lateral view **B** genitalia, lateral view. Abbreviations: C – cercus; H – hypandrium; LP – lateral process; PH – phallus; SDL – surstylus dorsal lobe; SVL – surstylus ventral lobe. Scale bars: 1 mm (**A**); 0.1 mm (**B**).

**Male** (Fig. 1A). Body length 5.0–5.7 mm, wing length 4.1–4.2 mm.

***Head*** metallic green with pale gray pollinosity; face very narrow, eyes contiguous on face. Hairs and bristles on head black, but postocular bristles (except uppermost two bristles) and postero-ventral hairs pale yellow. Antenna (Fig. 8A) yellow except first flagellomere brown; first flagellomere somewhat oval, approximately as long as wide, obtuse apically, with pale white pubescence; arista subapical, dark brown. Proboscis pale yellow with pale hairs; palpus pale yellow with pale yellow hairs and two to three short black apical bristles.

***Thorax*** dark yellow with fine pale yellow pollinosity; mesonotum dark yellow; postalar callus with a small dark brown spot; postnotum dark brown posteriorly. Pteropleuron with a small black subtriangular spot. Laterotergite with two-minute dark brown or black spots at anterior margin and one large dark brown spot posteriorly. Hairs and bristles on thorax black, six strong dc, 18–20 irregularly paired acr short hair-like; scutellum with two pairs of bristles, lateral pair long and strong, median pair short and hair-like. Propleuron with one brown bristle on lower portion.

***Legs*** mainly yellow, but fore tarsomeres 2–5 brown and mid and hind tarsomeres 2–5 dark brown. Hairs and bristles on legs black. Fore coxa with three or four thick bristles on antero-apical portion mostly or wholly dark yellow; mid coxa with three anterior and apical bristles; hind coxa with one strong outer bristle at basal 1/3. Mid and hind trochanters each with one outer bristle at middle. Fore tibia devoid of bristles; mid tibia with two ad, two pd and two apical bristles; hind tibia with three ad, four pd, three apical bristles and one row of brownish yellow comb-like bristles. Fore tarsomere 1 with two short thin apical bristles. Mid tarsomere 1 with five short or long apical bristles. Hind tarsomere 1 with cluster of short thick posterior bristles at extreme base, several short ventral bristles and four short apical bristles. Relative lengths of tibiae and five tarsomeres of legs –LI 5.3: 6.2: 3.2: 2.1: 1.9: 0.9; LII 5.9: 9.1: 2.9: 2.1: 1.2: 0.7; LIII 9.8: 4.8: 4.1: 2.2: 1.2: 0.8.

***Wing*** nearly hyaline, tinged brown; veins brown, M_1+2_ gently bent apically and convergent with R_4+5_; CuAx ratio 0.34. Squama yellow with pale yellow hairs. Halter yellow.

***Abdomen*** yellow with yellow pollen, terga 2–5 dark yellow; terga 2–5 each with large blackish spot somewhat narrowed posteriorly. Hairs and bristles on abdomen black except those on venter more or less yellow.

***Male genitalia*** (Fig. 1B) mainly shiny black. Epandrium longer than wide, with two lateral processes (one short and thin, with two apical hairs; the other long and wide, subtriangular). Ventral surstylus rather wide, widened apically; dorsal surstylus rather wide, slightly narrower than ventral surstylus, with very narrow medial incision at tip. Cercus somewhat round, white, bearing short white hairs. Hypandrium long and somewhat thick. Phallus thin, hidden within hypandrium.

**Female.** Unknown.

##### Distribution.

China (Yunnan).

##### Etymology.

The specific name refers to the ventral surstylus widened apically.

##### Remarks.

The species is very similar to *N.qingchengshana* Yang & Saigusa, 2021 from Sichuan, but may be separated from the latter by the fore tarsomere 1 longer than fore tibia and ventral surstylus much widened at extreme tip. In *N.qingchengshana*, the fore tarsomere 1 is as long as the fore tibia, and the ventral surstylus is weakly widened at extreme tip (Yang and Saigusa 2001a; Yang et al. 2011).

#### ﻿ Neurigona basicurva sp. nov.

Taxon classificationAnimaliaDipteraDolichopodidae

3F1F6364-8C01-566B-B3DA-789B661040C2

https://zoobank.org/7B2FCEBC-C157-41BE-A196-16FD73B9979A

Figs 2 , 8

##### Type material.

***Holotype***: ♂, **China**: Yunnan, Lvchun, Yakou, Shuikuxiafang, (23°3'25.2"N, 102°46'37.71"E), 1780 m, collected by Malaise trap, 2019.IV.19-V.19, Liang Wang and Xin Li (CAU). ***Paratypes***: 2 ♂♂, the same data as holotype (CAU).

##### Diagnosis.

Mesonotum with three brown longitudinal stripes at middle posterior region. Abdominal sternite 5 quadrate. Fore tibia basally weakly thickened and bent. Ventral surstylus wide, distinctly bent with distinctly bent and narrowed tip; dorsal surstylus short and broad, folded inward, apically furcated.

##### Description.

**Figure 2.**
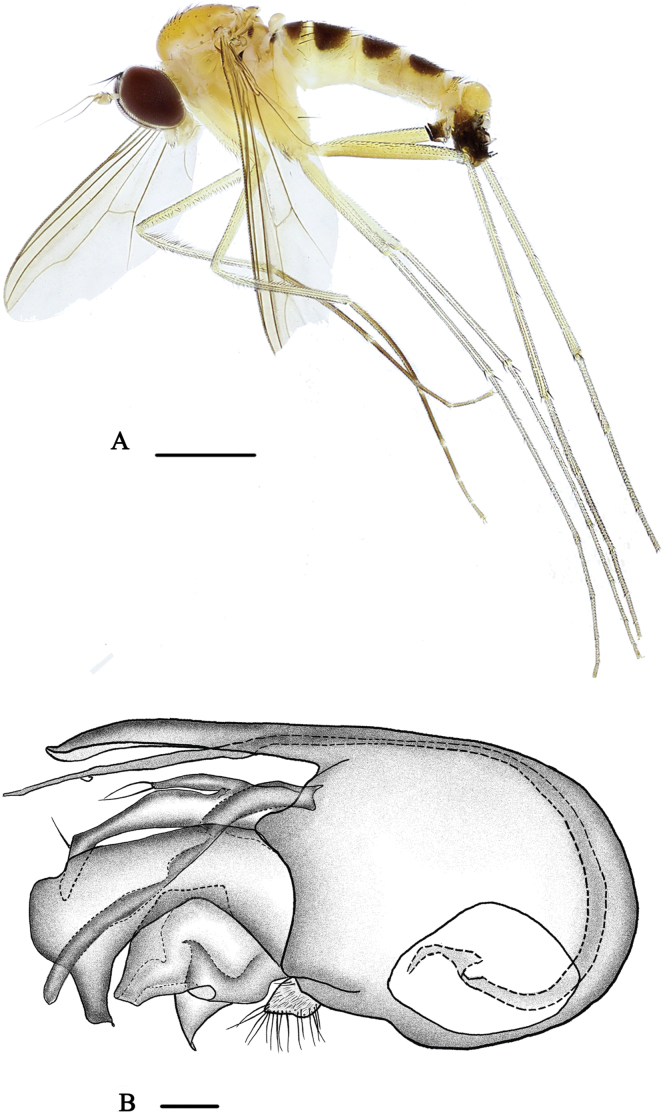
*Neurigonabasicurva* sp. nov. **A** male habitus, lateral view **B** genitalia, lateral view. Scale bars: 1 mm (**A**); 0.1 mm (**B**).

**Male** (Fig. 2A). Body length 3.8 mm, wing length 3.2 mm.

***Head*** metallic green with pale yellow pollinosity; eyes narrowly separated on middle portion of face. Hairs and bristles on head black, but postocular bristles and postero-ventral hairs pale yellow. Antennal scape and pedicel (Fig. 8B) yellow; first flagellomere dark yellow, almost as long as wide, round apically, with pale brown pubescence; arista subapical, brown, basal segment 0.1× longer than apical segment. Proboscis yellow with pale hairs; palpus pale yellow with two black apical bristles.

***Thorax*** yellow with fine pale yellow pollinosity; mesonotum dark yellow with three brown longitudinal stripes at middle posterior region; scutellum brownish; postnotum brownish with dark yellow basal margin; laterotergite with a small black inner spot at anterior margin. Pteropleuron below wing base with a small black spot. Hairs and bristles on thorax black, six strong dc, 11–12 irregularly paired acr short hair-like; scutellum with two pairs of bristles, lateral pair long and strong, median pair short and hair-like.

***Legs*** mainly yellow, but fore tarsus dark brown, mid and hind tarsi pale brown. Hairs and bristles on legs black. Fore coxa with six mostly yellow bristles on antero-apical portion; mid coxa with three anterior and apical bristles; hind coxa with one strong outer bristle at basal 1/3. Mid trochanter with two bristles, hind trochanter with one bristle. Fore femur with two rows of short dense av. Fore tibia modified, basally weakly thickened and bent, with two rows of long dense black anterior bristles at basal 1/3 and two rows of short dense brownish yellow anterior hairs at remaining 2/3. Mid tibia with one outer bristle and one pd, apically with one bristle. Hind tibia with one pd, apically with three bristles and one row of pale brown comb-like bristles apically. Fore tarsomere 1 modified, weakly bent, with short dense and erect pv; tarsomeres 2–4 with fine but dense ventral hairs. Mid tarsomere 1 with one pv at extreme base. Hind tarsomere 1 with cluster of short erect bristles basally. Relative lengths of tibiae and five tarsomeres of legs – LI 6.7: 5.9: 2.8: 1.8: 1.2: 1.0; LII 7.5: 8.8: 3.0: 2.0: 1.1: 0.8; LIII 12.1: 4.0: 4.0: 2.1: 1.3: 0.7.

***Wing*** nearly hyaline, tinged brown; veins brown, M_1+2_ gently bent apically and convergent with R_4+5_; CuAx ratio 0.45. Squama yellow, but brown at margin, with yellow hairs. Halter dark pale yellow.

***Abdomen*** yellow with pale yellow pollen; terga 1–4 each with large dark brown spot; sternum 5 projected, quadrate (Fig. 9A). Hairs and bristles on abdomen black except those on venter more or less yellow.

***Male genitalia*** (Fig. 2B): epandrium longer than wide, with two lateral processes (one very long and narrow; the other thick, with finger-like outer process near base, apically somewhat bent, and with one yellow bristle at apical 1/4). Ventral surstylus wide, distinctly bent with distinctly bent and narrowed tip; dorsal surstylus short and broad, folded inward, apically furcated. Cercus relatively small, trapezoid, white, with short pale yellow hairs. Hypandrium long and somewhat thick. Phallus long and thin, subapically with a denticle.

**Female.** Unknown.

##### Distribution.

China (Yunnan).

##### Etymology.

The specific name refers to the fore tibia basally weakly thickened and bent.

##### Remarks.

The species is peculiar and can be easily separated from other known species of the genus by the quadrate 5^th^ abdominal sternite and fore tibia basally weakly thickened and bent.

#### ﻿ Neurigona brevidigitata sp. nov.

Taxon classificationAnimaliaDipteraDolichopodidae

C62F13C7-3095-5459-90FF-B455718757CF

https://zoobank.org/E0876F26-7306-4292-9373-9109AC48E7EA

Figs 3 , 8

##### Type material.

***Holotype***: ♂, **China**: Yunnan, Lvchun, Yakou, Shuikuxiafang, (23°3'25.2"N, 102°46'37.71"E), 1780 m, collected by Malaise trap, 2019.IV.19-V.19, Liang Wang and Xin Li (CAU). ***Paratypes***: 69 ♂♂, 19 ♀♀, the same data as holotype (CAU).

##### Diagnosis.

Eyes almost contiguous on middle portion of face. Mesonotum with a brown mid-posterior stripe. Postnotum with a nearly W-shaped dark brown spot. Mid tibia and tarsus with row of short dense crochet-like av hairs. Hind tibia with short blackish comb-like apical bristles on very short and plate-like process. Ventral surstylus rather large and broad, ~ 1.5× wider than dorsal surstylus, apically with four long hairs.

##### Description.

**Figure 3.**
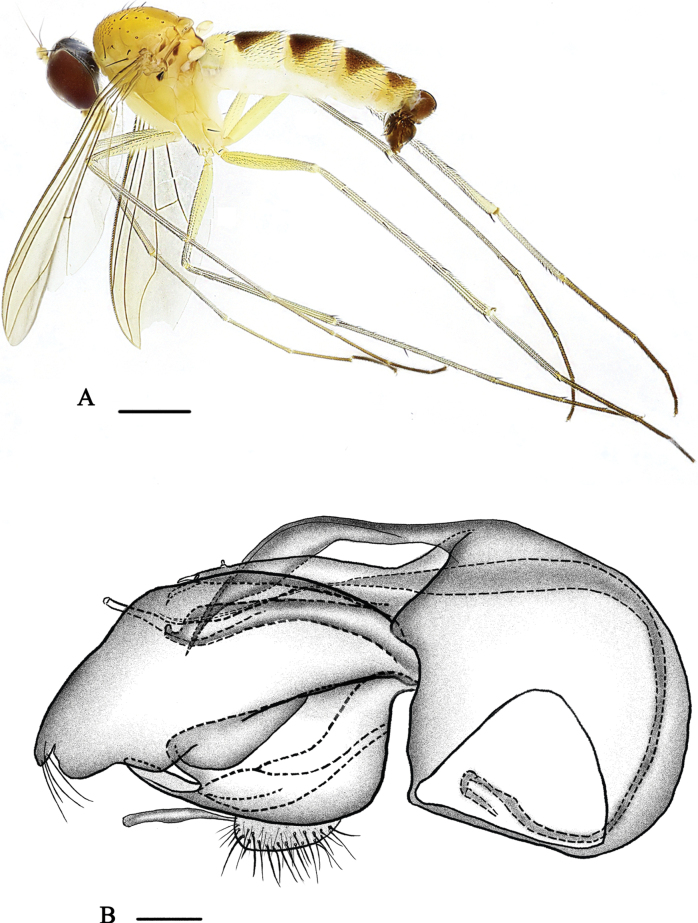
*Neurigonabrevidigitata* sp. nov. **A** male habitus, lateral view **B** genitalia, lateral view. Scale bars: 1 mm (**A**); 0.1 mm (**B**).

**Male** (Fig. 3A). Body length 5.4 mm, wing length 4.2 mm.

***Head*** metallic green with pale yellow pollinosity; eyes almost contiguous on middle portion of face. Hairs and bristles on head black but postocular bristles and postero-ventral hairs pale yellow. Antenna (Fig. 8C) yellow; first flagellomere somewhat oval, ~ 1.1× as long as wide, round apically, with brown pubescence; arista subapical, brownish. Proboscis yellow with pale hairs, with two long pale hairs, longer than proboscis; palpus pale yellow, but brownish at basal 1/3, with yellow hairs.

***Thorax*** yellow with fine pale yellow pollinosity; mesonotum with one brown longitudinal stripe at middle posterior region; scutellum brown at margin, with minute brown middle spot at basal margin; postnotum with a nearly W-shaped dark brown spot. Pteropleuron below wing base with a small black spot. Laterotergite with one blackish stripe and one minute brown spot at anterior margin. Hairs and bristles on thorax black; six strong dc, 12 irregularly paired acr short and hair-like; scutellum with two pairs of bristles, lateral pair long and strong, median pair short and hair-like. Propleuron with one yellow bristle on lower portion.

***Legs*** mainly yellow; fore tarsomeres 3–5 and mid and hind tarsomeres 2–5 dark brown. Hairs and bristles on legs black except hairs on fore coxa yellow. Fore coxa with four thick dark brownish yellow bristles on antero-apical portion; mid coxa with three or four partly brownish yellow or black anterior and apical bristles; hind coxa with one strong black outer bristle at basal 1/3. Mid and hind trochanters each with one outer bristle at middle. Fore tibia devoid of bristles. Mid tibia with two ad, two pd, row of short dense crochet-like av hairs, and two apical bristles. Hind tibia much elongated, 1.7× longer than fore tibia, with three ad, three pd, seven short thin pv, two thick apical bristles, and short blackish comb-like apical bristles on very short plate-like process. Fore tarsomeres 4 and 5 with slightly long hairs. Mid tarsus with row of short dense crochet-like av hairs; tarsomere 1 with two short ad, one long pd at extreme base, five av, and three apical bristles. Hind tarsomere 1 with pale-colored semi-fan-shaped ventral ridge bearing three short and three long hairs at extreme base, three av, and four apical bristles. Relative lengths of tibiae and five tarsomeres of legs – LI 6.8: 4.9: 3.2: 2.9: 2.1: 0.8; LII 8.2: 8.2: 3.5: 2.2:1.2: 0.7; LIII 11.5: 4.7: 4.1: 2.4: 1.3: 0.9.

***Wing*** nearly hyaline, tinged brown; veins brown, M_1+2_ gently bent apically and convergent with R_4+5_; CuAx ratio 0.16. Squama yellow but brown at margin, with yellow hairs. Halter dark yellow, with cluster of hairs at base of knob.

***Abdomen*** yellow with pale yellow pollen; terga 2–5 each with dark brown antero-laterally; hypopygium shiny dark brown. Hairs and bristles on abdomen black except those on venter more or less yellow.

***Male genitalia*** (Fig. 3B): epandrium nearly as long as wide, with two lateral processes (one long, somewhat thick, apically with two short or long and thin processes, and one denticle at apical 1/6; the other very long and thin. Ventral surstylus rather large and broad, ~ 1.5× wider than dorsal surstylus, apically with four long hairs; dorsal surstylus short and broad, apically folded with an oblique finger-like process. Cercus basally somewhat round, white, bearing short white hairs, apically long finger-like. Hypandrium quite long and thin, apically acute. Phallus rather long and thin, apically acute, hidden within hypandrium.

**Female.** Body length 4–5.2 mm, wing length 3.8–4.2 mm. Similar to male, except the following features: eyes distinctly separated on face, narrowest distance in middle of face equal to distance between ocellar bristles; postnotum with a nearly U-shaped dark brown spot. Relative lengths of tibiae and five tarsomeres of legs – LI 4.2: 3.3: 2.1: 1.7: 0.9: 0.6; LII 5.2: 2.8: 2.0: 1.2: 0.7: 0.5; LIII 8.0: 3.1: 2.6: 1.5: 1.0: 0.6.

##### Distribution.

China (Yunnan).

##### Etymology.

The specific name refers to the dorsal surstylus with a short finger-like process apically.

##### Remarks.

The species is very similar to *N.centralis* Yang & Saigusa from Yunnan, but may be separated from the latter by the postnotum yellow with a nearly W-shaped dark brown spot and the mid tibia and tarsus with row of short dense crochet-like av hairs. In *N.centralis*, the postnotum is wholly blackish, and the mid tibia and tarsus do not have the crochet-like av hairs except the mid tarsomere 1 with row of erect av hairs (Yang and Saigusa 2001b; Yang et al. 2011).

#### ﻿ Neurigona centralis

Taxon classificationAnimaliaDipteraDolichopodidae

Yang & Saigusa, 2001

351042BA-D52B-5572-A96A-3DF5B1A36157

Neurigona centralis Yang & Saigusa, 2001: 173. Type locality: China: Yunnan, Lijiang, Yulongxueshan.

##### Diagnosis.

Mesonotum with a black mid-posterior stripe. Postnotum wholly blackish. Mid tibia and tarsus without row of short dense crochet-like av hairs, but mid tarsomere 1 with row of erect av hairs. Epandrium short and wide. Dorsal surstylus short and wide with bifurcated apical process; ventral surstylus long, rather narrow.

##### Distribution.

China (Yunnan).

#### ﻿ Neurigona convexa sp. nov.

Taxon classificationAnimaliaDipteraDolichopodidae

60D11D5E-F7F1-5EF1-AEEA-6752AEC39C2F

https://zoobank.org/5591CEE2-411E-4518-9D3A-50885588E162

Figs 4 , 8

##### Type material.

***Holotype***: ♂, **China**: Yunnan, Lvchun, Yakou, Shuikuxiafang, (23°3'25.2"N, 102°46'37.71"E), 1780 m, collected by Malaise trap, 2019.IV.19-V.19, Liang Wang and Xin Li (CAU). ***Paratypes***: 3 ♂♂, the same data as holotype (CAU).

##### Diagnosis.

First flagellomere somewhat triangular. Wing subapically convex at anterior margin. Fore tarsomere 4 ventrally weakly swollen at extreme base and then weakly concave for a short distance. Ventral surstylus distinctly widened subapically, apically nearly finger-like; dorsal surstylus nearly as long as ventral surstylus, rather wide, apically somewhat narrow.

##### Description.

**Figure 4.**
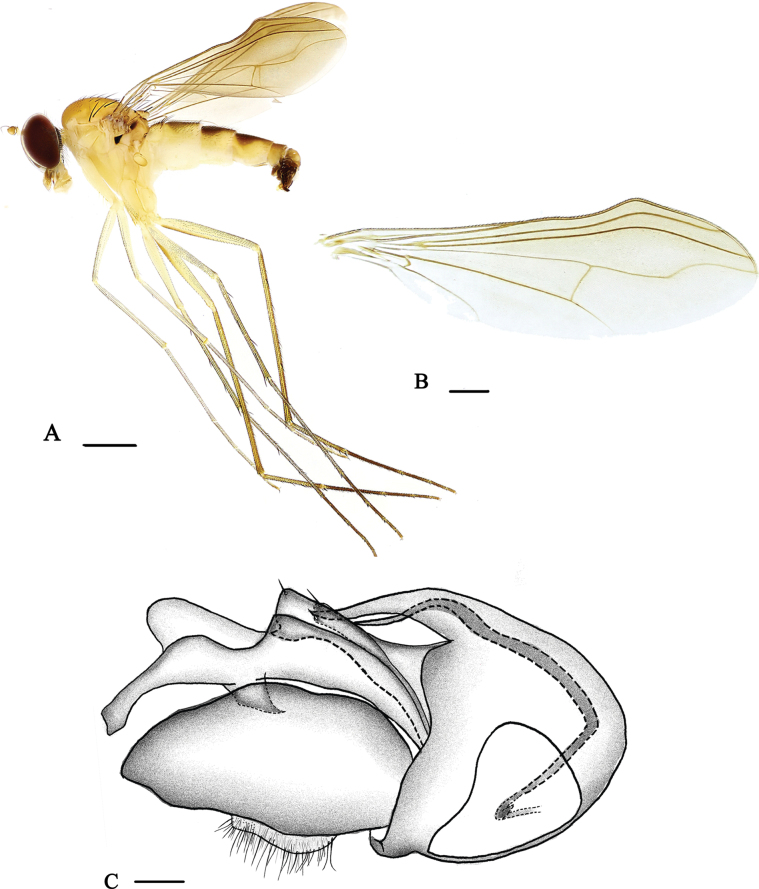
*Neurigonaconvexa* sp. nov. **A** male habitus, lateral view **B** wing **C** genitalia, lateral view. Scale bars: 1 mm (**A**); 0.05 mm (**B**); 0.1 mm (**C**).

**Male** (Fig. 4A). Body length 5.1–5.9 mm, wing length 5.0–5.7 mm.

***Head*** metallic green with pale gray pollinosity; eyes very narrowly separated on middle portion of face. Hairs and bristles on head black, but postocular bristles and postero-ventral hairs pale yellow. Antenna (Fig. 8D) pale yellow, pedicel yellow; first flagellomere dark brown, nearly triangular, almost as long as wide; arista subapical, dark brown, with short brown pubescence. Proboscis yellow with pale hairs; palpus yellow, but brown basally, with blackish setulae and two blackish setae.

***Thorax*** yellow with fine pale yellow pollinosity; mesonotum dark brownish yellow, but brown on flat mid-posterior portion; scutellum dark brownish yellow; postnotum dark brown at basal margin; laterotergite with a small black stripe at anterior margin. Pteropleuron with a very small black subtriangular spot just below wing base. Hairs and bristles on thorax black, six strong dc, 10–11 irregularly paired acr, short, hair-like; scutellum with two pairs of bristles, lateral pair long and strong, median pair short and hair-like. Propleuron with one brown bristle on lower portion.

***Legs*** mainly yellow, but mid and hind tibiae and tarsi brown to dark brown. Hairs and bristles on legs mostly black except hairs on fore coxa pale yellow. Fore coxa with five brownish bristles on antero-apical portion; mid coxa with three brownish anterior and apical bristles; hind coxa with one strong black outer bristle at basal 1/3. Fore tibia with one short ad. Mid tibia with three ad, two pd, and two black strong apical bristles. Hind tibia with three ad, three pd, three short apical bristles, and one row of brownish yellow comb-like bristles. Fore tarsus with very short hairs. Fore tarsomere 4 ventrally weakly swollen at extreme base and weakly incised outward, and with short and nearly erect ventral hairs; tarsomere 5 ventrally with short and nearly erect ventral hairs. Mid tarsomere 1 with two short ad, three short av, and four short thick apical bristles; tarsomeres 2–4 with several short spine-like ventral bristles. Hind tarsomere 1 with thick ventral bristles and three short thick apical bristles. Relative lengths of tibiae and five tarsomeres of legs LI – 7.1: 5.9: 2.2: 1.4:1.1: 0.9; LII 8.1: 8.2: 2.2: 1.3: 0.9: 0.8; LIII 11.2: 3.8: 3.6: 1.5: 1.2: 0.7.

***Wing*** (Fig. 4B) subapically convex forward at anterior margin; nearly hyaline, tinged brown; veins brown; R_2+3_ and R_4+5_ convex forward at apical 1/3 and somewhat parallel; M_1+2_ gently bent apically and convergent with R_4+5_; CuAx ratio 0.75. Squama brown with pale yellow hairs. Halter dark yellow with brown knob.

***Abdomen*** yellow with pale yellow pollen; terga 2–5 each with large dark brown spot at middle; hypopygium shiny dark brown. Abdominal sternite 5 distinctly projected, large subtriangular (Fig. 9B). Hairs and bristles on abdomen blackish except those on venter more or less yellow.

***Male genitalia*** (Fig. 4C): epandrium wider than long, with two lateral processes (one long finger-like; the other long and somewhat thick, apically slightly dilated with very short and acute apico-dorsal process). Ventral surstylus distinctly widened subapically, apically nearly finger-like; dorsal surstylus nearly as long as ventral surstylus, rather wide, apically somewhat narrow. Cercus somewhat round, white, bearing short pale yellow hairs. Hypandrium short and acute. Phallus somewhat short and thin, hidden with hypandrium.

**Female.** Unknown.

##### Distribution.

China (Yunnan).

##### Etymology.

The specific name refers to the wing subapically convex at anterior margin.

##### Remarks.

The new species is peculiar and can be easily separated from other known species of the genus by the wing subapically convex at anterior margin and arista shorter than head width.

#### ﻿ Neurigona huanglianshana sp. nov.

Taxon classificationAnimaliaDipteraDolichopodidae

BDAA6775-5A77-5C16-BD13-5739D9A54168

https://zoobank.org/1E16CFD0-258E-4930-A199-99719DED5F27

Figs 5 , 8

##### Type material.

***Holotype***: ♂, **China**: Yunnan, Lvchun, Yakou, Shuikuxiafang, (23°3'25.2"N, 102°46'37.71"E), 1780 m, collected by Malaise trap, 2019.IV.19-V.19, Liang Wang and Xin Li (CAU).

##### Diagnosis.

Mesonotum with brown subtriangular spot at middle posterior region; scutellum dark brownish yellow with dark yellow posterior margin; postnotum dark brown with dark yellow anterior margin. First flagellomere somewhat oval. Hind tarsomere 1 with cluster of short, erect, and fine ventral bristles basally, three pv on apical 1/3, and two apical bristles. Ventral surstylus apically strongly bent, with bifurcated tip; dorsal surstylus wider than ventral surstylus, nearly quadrate, apically with one knife-like dorsal process.

##### Description.

**Figure 5.**
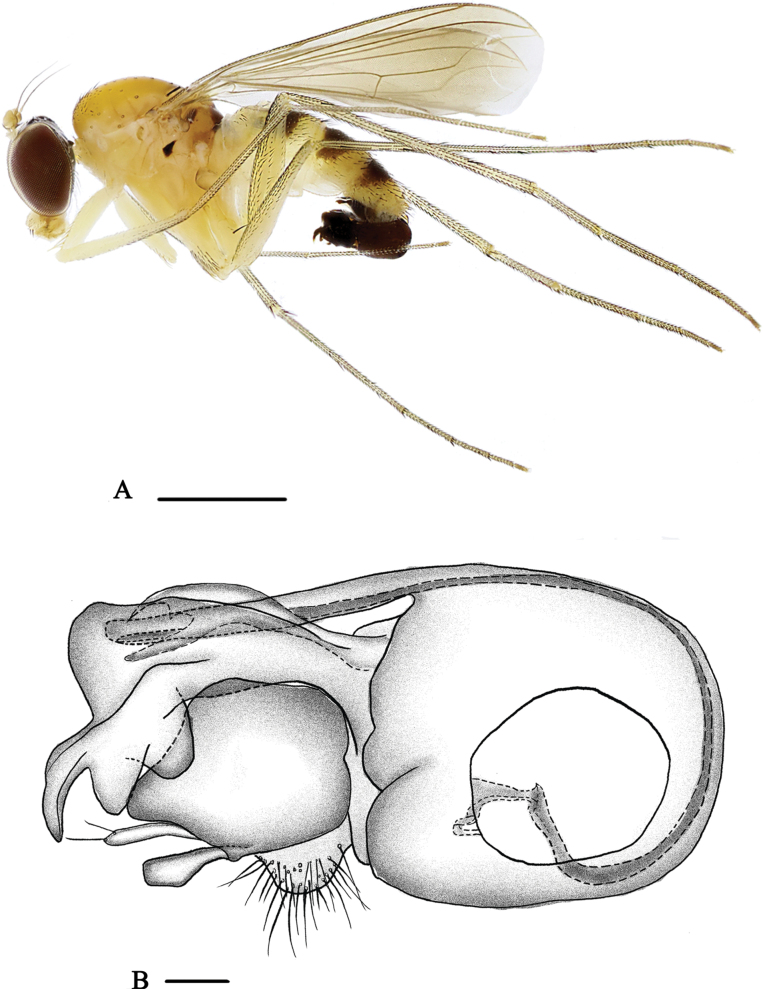
*Neurigonahuanglianshana* sp. nov. **A** male habitus, lateral view **B** genitalia, lateral view. Scale bars: 1 mm (**A**); 0.1 mm (**B**).

**Male** (Fig. 5A). Body length 3.0 mm, wing length 3.5 mm.

***Head*** metallic green with gray pollinosity; eyes very narrowly separated on middle portion of face. Hairs and bristles on head black, but postocular bristles and postero-ventral hairs pale yellow. Antenna (Fig. 8E) yellow except first flagellomere dark yellow; first flagellomere somewhat oval, almost as long as wide, somewhat round apically, with dense brownish pubescence; arista subapical, dark brown with dark brownish yellow base. Proboscis yellow with pale bristles and hairs; palpus yellow with two strong black apical bristles.

***Thorax*** yellow with fine pale yellow pollinosity; mesonotum with brown subtriangular spot at middle posterior region; scutellum dark brownish yellow with dark yellow posterior margin; postnotum dark brown with dark yellow anterior margin; laterotergite with blackish stripe at anterior margin and dark brown inner portion. Pteropleuron below wing base with a small black spot. Hairs and bristles on thorax black, six or seven strong dc, 10–12 irregularly paired acr short hair-like; scutellum with two pairs of bristles, lateral pair long and strong, median pair short and hair-like. Propleuron with one yellow bristle on lower portion.

***Legs*** yellow except fore tarsus dark yellow with tarsomere 5 brown and mid and hind tarsi brownish yellow. Hairs and bristles on legs mostly black except hairs on fore coxa brownish yellow to brown. Fore coxa with four long thick brownish yellow bristles on antero-apical portion; mid coxa with three brownish yellow anterior and apical bristles; hind coxa with one strong black outer bristle at basal 1/3. Mid tibia with one ad, one pd, and two short or long apical bristles. Hind tibia with three ad, two pd, two apical ventral bristles, and one row of brownish yellow comb-like bristles. Mid tarsomere 1 with one short and one long dorsal bristles at base. Hind tarsomere 1 with cluster of short erect and fine ventral bristles basally, three pv on apical 1/3, and two apical bristles. Relative lengths of tibiae and five tarsomeres of legs – LI 11.3: 8.0: 5.0: 3.3:1.9: 1.0; LII 13.4: 14.1: 3.7: 2.9:1.6: 2.4; LIII 20.6: 5.9: 6.3: 3.8: 2.7: 1.2.

***Wing*** nearly hyaline, tinged brown; veins brown, M_1+2_ distinctly bent, somewhat geniculate, greater than 90 degrees; CuAx ratio 0.49. Squama yellow with yellow hairs. Halter yellow, but base of knob brown with cluster of black hairs.

***Abdomen*** yellow with pale yellow pollen; terga 2–4 each with dark brown basal spot; abdominal sternum 5 distinctly projected, long and subtriangular (Fig. 9C). Hairs and bristles on abdomen black except those on venter more or less yellow.

***Male genitalia*** (Fig. 5B): epandrium almost as long as wide, with two lateral processes (one extremely long and thin; the other long, somewhat wide, apically furcated). Ventral surstylus apically strongly bent, with bifurcated tip; dorsal surstylus wider than ventral surstylus, nearly quadrate, apically with one knife-like dorsal process. Cercus round with long dense yellow hairs, with long finger-like apical process. Hypandrium long and narrow. Phallus long and thin.

**Female.** Unknown.

##### Distribution.

China (Yunnan).

##### Etymology.

The species is named after the type locality Huanglianshan.

##### Remarks.

This species is somewhat similar to *N.henana* Wang, Yang & Grootaert, 2007 from Henan of China, but may be separated from the latter by the arista subapical and mesonotum with one brown subtriangular spot at middle posterior region. In *N.henana*, the arista is dorsal, and the mesonotum is wholly yellow (Wang et al. 2007; Yang et al. 2011).

#### ﻿ Neurigona quadrimaculata sp. nov.

Taxon classificationAnimaliaDipteraDolichopodidae

00A031F2-B9CF-58DD-A18B-8F5440CA3CE0

https://zoobank.org/65153364-6CA4-4CD4-B4A5-B7B4CFD5CBA7

Figs 6 , 8

##### Type material.

***Holotype***: ♂, **China**: Yunnan, Lvchun, Yakou, Shuikuxiafang, (23°3'25.2"N, 102°46'37.71"E), 1780 m, collected by Malaise trap, 2019.IV.19-V.19, Liang Wang and Xin Li (CAU). ***Paratypes***: 21 ♂♂, the same data as holotype (CAU).

##### Diagnosis.

Eyes contiguous on face. Postnotum and laterotergite dark brown. Thoracic pleuron with four dark spots. Mid tarsus with row of crochet-like av hairs. Ventral surstylus longer than dorsal surstylus, rather narrow, but wide at apex; dorsal surstylus very wide, ~ 3× wider than ventral surstylus, with a long acute apico-dorsal process.

##### Description.

**Figure 6.**
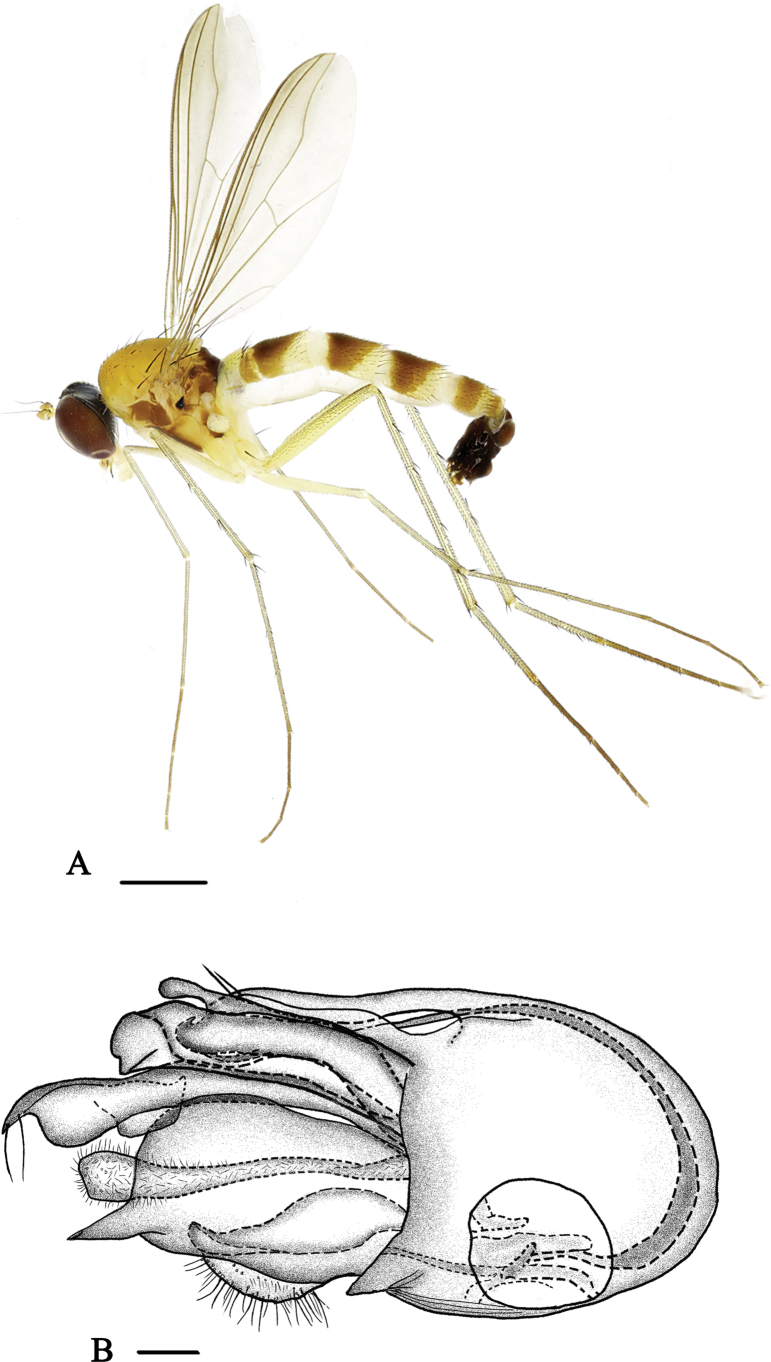
*Neurigonaquadrimaculata* sp. nov. **A** male habitus, lateral view **B** genitalia, lateral view. Scale bars: 1 mm (**A**); 0.1 mm (**B**).

**Male** (Fig. 6A). Body length 5.2–6.1 mm, wing length 4.6–4.9 mm.

***Head*** metallic green with pale gray pollinosity; face very narrow, eyes contiguous on face. Hairs and bristles on head black, but postocular bristles (except uppermost two) and postero-ventral hairs yellow. Antenna (Fig. 8F) yellow except first flagellomere brownish; first flagellomere basally wide, apically narrowed and obtuse, approximately as long as wide, with brown pubescence; arista subapical, dark brown. Proboscis pale yellow with dark yellow hairs; palpus pale yellow with blackish hairs and two short black apical bristles.

***Thorax*** dark yellow with fine pale yellow pollinosity; mesonotum wholly dark yellow, postalar callus with a small dark brown spot; scutellum yellow; postnotum and laterotergite dark brown; mesopleuron, sternopleuron (except posterior portion), and hypopleuron (except postero-dorsal corner) dark brown, pteropleuron brown with a small black spot. Hairs and bristles on thorax black, six strong dc gradually becoming longer backward, 11–12 irregularly paired acr short and hair-like; scutellum with two pairs of sc, lateral pair long and strong, median pair very short and weak. Propleuron with one brown bristle on lower portion.

***Legs*** mainly yellow, but brown or dark brown from tip of tarsomere 1 onward. Hairs and bristles on legs black except hairs on fore coxa yellow. Fore coxa with four thick yellow bristles on antero-apical portion; mid coxa with three mostly dark yellow anterior and apical bristles; hind coxa with one strong black outer bristle at basal 1/3. Mid and hind trochanters each with one spine-like outer bristle at middle. Fore tibia devoid of bristles. Mid tibia with three ad, two pd, and two strong apical bristles. Hind tibia with three ad, three pd, three strong apical bristles, and one row of yellow comb-like bristles. Mid tarsus with row of crochet-like av hairs; tarsomere 1 with one short ad at middle and one long pd at extreme tip. Relative lengths of tibiae and five tarsomeres of legs – LI 5.5: 5.9: 3.2: 2.3: 1.2: 0.8; LII 7.6: 7.9: 3.3: 1.8: 1.1: 0.8; LIII 11.2: 4.2: 4.0: 1.9: 1.2: 0.8.

***Wing*** nearly hyaline, tinged brown; veins brown, M_1+2_ gently bent apically and convergent with R_4+5_; CuAx ratio 0.29. Squama yellow with pale yellow hairs. Halter pale yellow.

***Abdomen*** yellow with yellow pollen; terga 2–5 each with large blackish basal spot. Hairs and bristles on abdomen chiefly black except those on venter more or less yellow.

***Male genitalia*** (Fig. 6B) mainly shiny black. Epandrium longer than wide, with short acute apico-dorsal corner, with three lateral processes, ventral one long and finger-like, middle one long and wide, apically with a nearly U-shaped incision; dorsal one long and wide, curly at tip. Ventral surstylus longer than dorsal surstylus, rather narrow, but wide at apex; dorsal surstylus very wide, ~ 3.0× wider than ventral surstylus, with a long acute apico-dorsal process. Postgonite long and thick, covered with fine hairs. Cercus somewhat round, white, bearing short white hairs. Hypandrium long and wide. Phallus thin, hidden within hypandrium.

**Female.** Unknown.

##### Distribution.

China (Yunnan).

##### Etymology.

The specific name refers to the thoracic pleuron with four dark spots.

##### Remarks.

The species is similar to *N.qingchengshana* Yang & Saigusa, 2021 from Sichuan, but may be separated from the latter by the fore tarsomere 1 longer than fore tibia, 1.1× as long as fore tibia; and narrow ventral surstylus. In *N.qingchengshana*, the fore tarsomere 1 is as long as the fore tibia, and the ventral surstylus is rather wide (Yang and Saigusa 2001a; Yang et al. 2011).

#### ﻿ Neurigona ventralis

Taxon classificationAnimaliaDipteraDolichopodidae

Yang & Saigusa, 2005

1E0E4BE4-5D3C-52D5-BDB3-CC4A485AB91C

Neurigona ventralis Yang & Saigusa, 2005: 759. Type locality: China: Shaanxi, Fuping.

##### Diagnosis.

Postnotum dark brown. First flagellomere brownish with yellow base. Fore tarsomeres 3 and 4 shortened, each 0.4× and 0.2× as long as tarsomere 2. respectively; tarsomeres 2 and 3 with long dorsal bristles, tarsomeres 3–5 with two rows of spine-like ventral bristles. Wing wholly hyaline. Dorsal surstylus short and wide with bifurcated apical processes; ventral surstylus long, rather narrow.

##### Distribution.

China (Yunnan, Shaanxi).

#### ﻿ Neurigona ventriprocessa sp. nov.

Taxon classificationAnimaliaDipteraDolichopodidae

31E40045-A31D-5ED5-9439-B21E7805468B

https://zoobank.org/8CC18FA7-1D35-48D6-9CB9-E548BE07A403

Figs 7 , 8

##### Type material.

***Holotype***: ♂, **China**: Yunnan, Lvchun, Yakou, Shuikuxiafang, (23°3'25.2"N, 102°46'37.71"E), 1780 m, collected by Malaise trap, 2019.IV.19-V.19, Liang Wang and Xin Li (CAU). ***Paratypes***: 3 ♂♂, the same data as holotype (CAU).

##### Diagnosis.

Frons and face yellow. First flagellomere somewhat quadrate. Mesonotum brownish to brown, but notopleuron somewhat pale; scutellum brown with dark yellow apical margin; postnotum brown with blackish middle line. Fore tarsomere 3 shortened and tarsomere 4 ventrally weakly concave with mammillary ventral process at extreme base. Ventral surstylus distinctly longer than dorsal surstylus, distinctly widened at middle, apically narrowed with short finger-like ventral process; dorsal surstylus short, much widened, ~ 1.5× wider than ventral surstylus.

##### Description.

**Figure 7.**
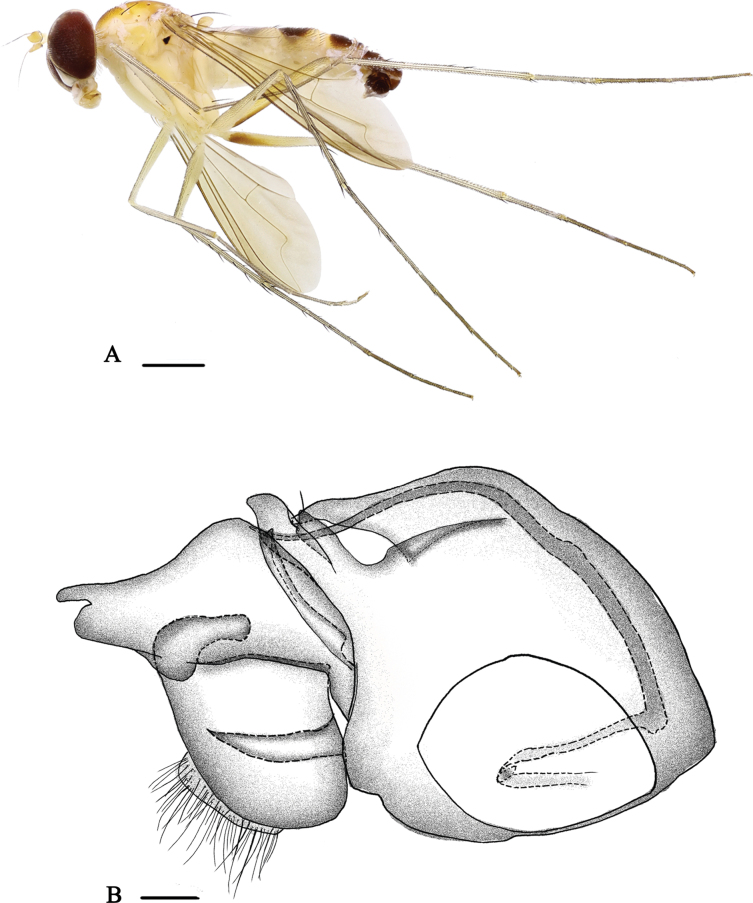
*Neurigonaventriprocessa* sp. nov. **A** male habitus, lateral view **B** genitalia, lateral view. Scale bars: 1 mm (**A**); 0.1 mm (**B**).

**Male** (Fig. 7A). Body length 6.9 mm, wing length 5.2 mm.

**Figure 8.**
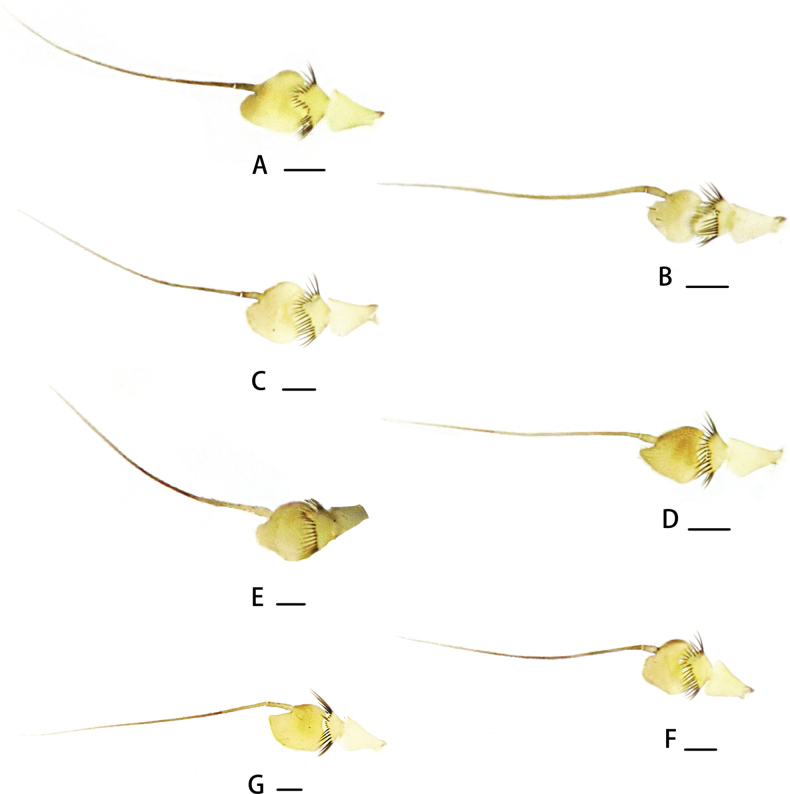
Antennae **A***Neurigonaapicilata* sp. nov. **B***Neurigonabasicurva* sp. nov. **C***Neurigonabrevidigitata* sp. nov. **D***Neurigonaconvexa* sp. nov. **E***Neurigonahuanglianshana* sp. nov. **F***Neurigonaquadrimaculata* sp. nov. **G***Neurigonaventriprocessa* sp. nov. Scale bars: 0.1 mm.

***Head*** metallic green with gray pollinosity, but yellow ventrally; eyes very narrowly separated on middle portion of face. Hairs and bristles on head black, but postocular bristles and postero-ventral hairs pale yellow. Antenna (Fig. 8G) pale yellow except first flagellomere brown; first flagellomere somewhat quadrate, 1.1× longer than wide, with slightly long, dense brownish pubescence; arista dorsal, dark brown. Proboscis mostly dark yellow, partly brown, with dark yellow and brown hairs; palpus dark yellow, but brown at base, with dark yellow hairs and two short brown bristles.

***Thorax*** mostly dark yellow to brownish yellow with fine pale yellow pollinosity; mesonotum brownish to brown, but notopleuron somewhat pale; scutellum brown with dark yellow apical margin; postnotum brown with blackish middle line. Laterotergite with blackish stripe at anterior margin. Pteropleuron with a small black subtriangular spot. Hairs and bristles on thorax black, five strong dc, 14–15 irregularly acr short hair-like; scutellum with two pairs of bristles, lateral pair long and strong, median pair short and hair-like. Propleuron with one brown bristle on lower portion.

***Legs*** mainly yellow, but hind femur ventrally dark brown at basal 1/3; mid and hind tarsomeres 2–5 brown or dark brown. Hairs and bristles on legs mostly black except hairs on all coxae dark yellow. Fore coxa with dark yellow hairs and six mostly brownish yellow bristles on antero-apical portion; mid coxa with two short black bristles and eight dense dark yellow anterior and apical bristles; hind coxa with dark yellow hairs and one strong outer black bristle at basal 1/3. Mid and hind trochanters each with one outer bristle at middle. All femora distinctly thickened basally. Fore tibia with one short ad and two short pd. Mid tibia with two ad, three pd, one av, two pv, and three apical bristles. Hind tibia with three ad, three pd, two short or long apical bristles, and one row of yellow comb-like bristles. Fore tarsomere 3 shortened with short dense ventral hairs; tarsomere 4 somewhat whitened, ventrally weakly concave, somewhat bare at middle, extreme base distinctly dilated with mammillary ventral process, extreme tip weakly dilated with several short curly brown ventral hairs; tarsomere 5 with quite dense, curly and dark brown ventral hairs. Mid tarsomere 1 with several short or long bristles. Relative lengths of tibiae and five tarsomeres of legs LI – 6.8: 6.7: 2.9: 0.8:1.1: 1.0; LII 8.8: 9.1: 3.7: 2.4:1.3: 0.8; LIII 13.9: 4.9: 4.8: 2.6: 1.8: 0.9.

***Wing*** nearly hyaline, tinged brown; veins dark brown, M_1+2_ strongly bent, geniculate nearly in a right angle; CuAx ratio 0.54. Squama dark yellow with brown margin bearing yellow hairs. Halter dark yellow with dark brown knob.

**Figure 9.**
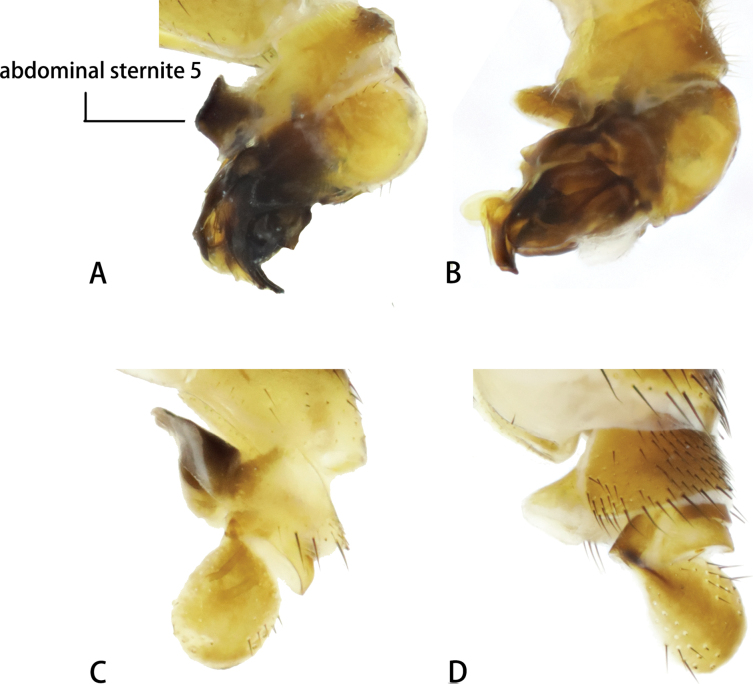
Abdominal sternite 5 (male) **A***Neurigonabasicurva* sp. nov. **B***Neurigonaconvexa* sp. nov. **C***Neurigonahuanglianshana* sp. nov. **D***Neurigonaventriprocessa* sp. nov.

***Abdomen*** yellow with pale yellow pollen; terga 1–4 each with large dark brown sport, tergum 5 wholly brown; sternum 5 weakly projected, small and subtriangular (Fig. 9D). Hairs and bristles on abdomen black except those on venter more or less yellow.

***Male genitalia*** (Fig. 7B): epandrium nearly as long as wide, with two lateral processes (one short finger-like; the other slightly long and thick, finger-like). Ventral surstylus distinctly longer than dorsal surstylus, distinctly widened at middle, apically narrowed with short finger-like ventral process; dorsal surstylus short, much widened, ~ 1.5× wider than ventral surstylus. Cercus long, with long dense yellow hairs. Hypandrium basally rather thick, apically narrowed. Phallus long and thin.

**Female.** Unknown.

##### Distribution.

China (Yunnan).

##### Etymology.

The specific name refers to the fore tarsomere 4 with mammillary ventral process at extreme base.

##### Remarks.

The species is somewhat similar to *N.guangxiensis* Yang, 1999 and *N.zhejiangensis* Yang, 1999 from Oriental China in having the nearly quadrate first flagellomere, but may be separated from them by the mesonotum brownish to brown, fore tarsomere 3 shortened and tarsomere 4 ventrally weakly concave with mammillary ventral process at extreme base. In *N.guangxiensis* and *N.zhejiangensis*, the mesonotum is yellow with markings on the middle posterior portion, and the fore tarsus is not modified (Yang et al. 2011).

#### ﻿ Neurigona yunnana

Taxon classificationAnimaliaDipteraDolichopodidae

Wang, Yang & Grootaert, 2007

0F887A65-DE7D-5C13-8E99-2586B61BBEC3

Neurigona yunnana Wang, Yang & Grootaert, 2007: 38. Type locality: China: Yunnan, Mengla.

##### Diagnosis.

Antenna brownish. Four strong dc; quadriseriate acr. Fore coxa with 4–6 black bristles on antero-apical portion. Apex of fore tarsomere 4 and entire fore tarsomere 5 white with white hairs. Hypopygium yellow. Dorsal surstylus wide with nearly acute tip; ventral surstylus slightly longer than dorsal surstylus, apically distinctly widened.

##### Distribution.

China (Yunnan).

## ﻿Discussion

The fauna of China has been divided into seven ecoregions: Northeast China, North China, Mongolia-Xinjiang Region, Qinghai-Tibet Regionthat belong to the Palaearctic Realm, and Southwest China, Central China, and South China Region that belong to the Oriental Realm (Zhang 1998). Based on the zoogeographical regions of China, eleven species (*N.centralis*, *N.qingchengshana*, *N.yunnana*, *N.sichuana*, *N.apicilata* sp. nov., *N.basicurva* sp. nov., *N.brevidigitata* sp. nov., *N.convexa* sp. nov., *N.huanglianshana* sp. nov., *N.quadrimaculata* sp. nov., *N.ventriprocessa* sp. nov.) are thus far only recorded from the Southwest China region, nine species (*N.composita*, *N.denudata*, *N.exemta*, *N.gemina*, *N.guangdongensis*, *N.guangxiensis*, *N.pectinata*, *N.xui*, *N.hainana*) are exclusively reported from the South China Region, eight species (*N.basalis*, *N.bimaculata*, *N.henana*, *N.micropyga*, *N.shaanxiensis*, *N.xiangshana*, *N.xiaolongmensis*) are only found in the North China Region, six species (*N.chetitarsa*, *N.guizhouensis*, *N.jiangsuensis*, *N.shennongjiana*, *N.wui*, *N.zhejiangensis*) are discovered in the Central China Region, two species (*N.grisea*, *N.yaoi*) are recorded from the Mongolia-Xinjiang Region, and one species (*N.xizangensis*) is exclusively found in the Qinghai-Tibet Region. Only two species, *N.concaviuscula* and *N.ventralis*, are common in the Central and Southwest China regions, and North and Southwest China regions respectively. Up to now, of the 38 species described from China, 30 species (79%) are recorded from Oriental China, while only eight species (21%) were recorded from Palaearctic China. Within the global zoogeographical realms, the distribution proportions of the *Neurigona* in the Palaearctic and Oriental realms are relatively equal, accounting for 27% and 23%, respectively. This forms a sharp contrast to its proportion in Oriental and Palaearctic China, indirectly suggesting that the current species richness of *Neurigona* in China is still underestimated. Further collections and investigations of *Neurigona* from more areas of China are needed to provide additional data on the distribution of this genus.

**Figure 10.**
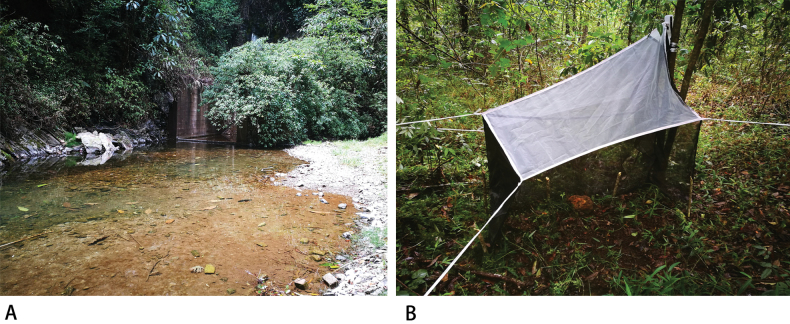
**A** forest habitat in Yunnan Province, Lvchun, Yakou, Shuikuxiafang, 1779 m **B** malaise trap set nearby.

The seven new species described in this study were collected by Malaise traps from the same locality, indicating a high level of local sympatry. There are many references indicating that the subfamily Medeterinae also exhibits rich local sympatry. For example, six species of *Systenus* were found from the same locality near Manaus, Brazil (Naglis 2000), eight species of *Systenus* were known only from Malaise traps at a single locality in Guanacaste Province, Costa Rica (Bickel 2015), and six species of *Systenus* were collected at the same collecting site in Yunnan, China (Lin et al. 2023). Furthermore, although *Neurigona* species can also be found in garden or park landscapes, they show a clear preference for wooded sites. The collection site of the material in this study precisely corresponds to such a habitat, which is located underneath an isolated area separated by a dam, with a stream running through the middle and a mixed environment of dense shrubland and broad-leaved forest on both sides (Fig. 10).

## Supplementary Material

XML Treatment for Neurigona apicilata

XML Treatment for Neurigona basicurva

XML Treatment for Neurigona brevidigitata

XML Treatment for Neurigona centralis

XML Treatment for Neurigona convexa

XML Treatment for Neurigona huanglianshana

XML Treatment for Neurigona quadrimaculata

XML Treatment for Neurigona ventralis

XML Treatment for Neurigona ventriprocessa

XML Treatment for Neurigona yunnana

## ﻿Additional information

### Author ORCIDs

Chen Lin https://orcid.org/0000-0002-9856-5012

Mengqing Wang https://orcid.org/0000-0001-8350-9301

Ding Yang https://orcid.org/0000-0002-7685-3478

### Data availability

All of the data that support the findings of this study are available in the main text.
